# Splenic Infarcts in a 22-Year-Old Boxer With Acute Epstein-Barr Virus Infection Without a Predisposing Haematological Disease

**DOI:** 10.7759/cureus.99062

**Published:** 2025-12-12

**Authors:** Shubham Gupta, Omur Choudhury

**Affiliations:** 1 General Internal Medicine, The Hillingdon Hospitals NHS Foundation Trust, London, GBR

**Keywords:** ebv positive, ebv splenomegaly, epstein-barr virus, glandular fever, splenic infarcts

## Abstract

Splenic infarction is a rare complication of Epstein-Barr virus (EBV) infectious mononucleosis, particularly in the absence of pre-existing haematological disease. We report a 22-year-old male with ulcerative colitis on azathioprine who presented with fever, sore throat, malaise, cervical lymphadenopathy and left upper quadrant pain. Imaging revealed splenomegaly with multiple wedge-shaped hypodense lesions consistent with splenic infarction. Laboratory investigations demonstrated marked lymphocytosis, reduced protein C, and a heterozygous Factor V Leiden mutation. EBV viral capsid antigen immunoglobulin M (IgM) was positive. The patient was managed conservatively with intravenous fluids, prophylactic anticoagulation and temporary suspension of azathioprine.

Splenic infarction is an uncommon but important manifestation of EBV-associated infectious mononucleosis. Postulated mechanisms include rapid splenic enlargement, transient hypercoagulability, and immunological processes. Conservative management is appropriate in most cases. Clinicians should maintain a high index of suspicion for splenic infarction in patients with infectious mononucleosis who report left upper quadrant pain. Early diagnosis is essential to prevent complications such as splenic rupture, and patients must be counselled to avoid contact sports during recovery.

## Introduction

Epstein-Barr virus (EBV) infection commonly presents with fever, lymphadenopathy and pharyngitis. Although splenomegaly is a frequent finding, splenic infarction is rarely reported, with fewer than 50 documented cases in the literature. The condition may be under-recognised due to limited imaging unless prompted by characteristic symptoms [1]. Proposed mechanisms include transient hypercoagulability, rapid splenic enlargement, and immunological activation [2-4]. We describe a case of splenic infarction in a young boxer with EBV infection and no known prior prothrombotic disorder.

## Case presentation

A 22-year-old, white male with a background of ulcerative colitis and well-controlled asthma presented to the Emergency Department with 9 days of pyrexia associated with rigours, chills and reduced appetite. He had a sore throat, intermittent cough, generalised malaise and lethargy, as well as three days of cervical lymphadenopathy. He reported 10 days of left upper quadrant pain, exacerbated by straining. From a colitis perspective, he had had 2 days of bloody diarrhoea followed by 2 days of non-bloody diarrhoea, which had subsequently settled (he had been on maintenance therapy of azathioprine 150 mg OD and Mesalazine 2 g BD with a previous flare six months prior to presentation that was treated successfully with an eight-week weaning regime of prednisolone). In addition, he had developed a vesicular rash on the right V1 dermatomal distribution five days prior to admission and had been commenced on Aciclovir four days prior for treatment of Varicella Zoster virus. He had no headache, vomiting or visual disturbance. There were no urinary symptoms or abdominal pain. Travel history was unremarkable, with no significant risks identified.

His observations were initially unremarkable (temperature: 36.3 °C, heart rate: 82 beats/minute, respiratory rate: 18 breaths/min, blood pressure: 123/68 mmHg, O2 saturation: 97% on room air).

Clinical examination revealed a crusted vesicular rash in the right forehead, non-tender cervical lymphadenopathy and a soft, mildly tender abdomen in the left upper quadrant with a palpable lower splenic border. Throat examination was unremarkable. Laboratory investigations on initial presentation revealed monocytosis, elevated liver enzymes and raised C-reactive protein (Table 1).

**Table 1:** Laboratory blood test results obtained on initial presentation

Laboratory test	Value	Reference range
lactate	1.5 mmol/L	<2 mmol/L
white cell count	9.3 x10^9^/L	4-11 x10^9^/L
haemoglobin	133 g/L	130-180 g/L
platelets	173 x10^9^/L	150-450 x10^9^/L
monocytes	1.1 x10^9^/L (elevated)	0.2-1.0 x10^9^/L
alanine aminotransferase	920 IU/L (elevated)	0-45 IU/L
alkaline phosphatase	296 IU/L (elevated)	30-130 IU/L
gamma-glutamyl transpeptidase	384 IU/L (elevated)	<55 IU/L
bilirubin	37 micromol/L (elevated)	0-21 micromol/L
C-reactive protein	103.2 mg/L (elevated)	0-5 mg/L

**Figure 1:**
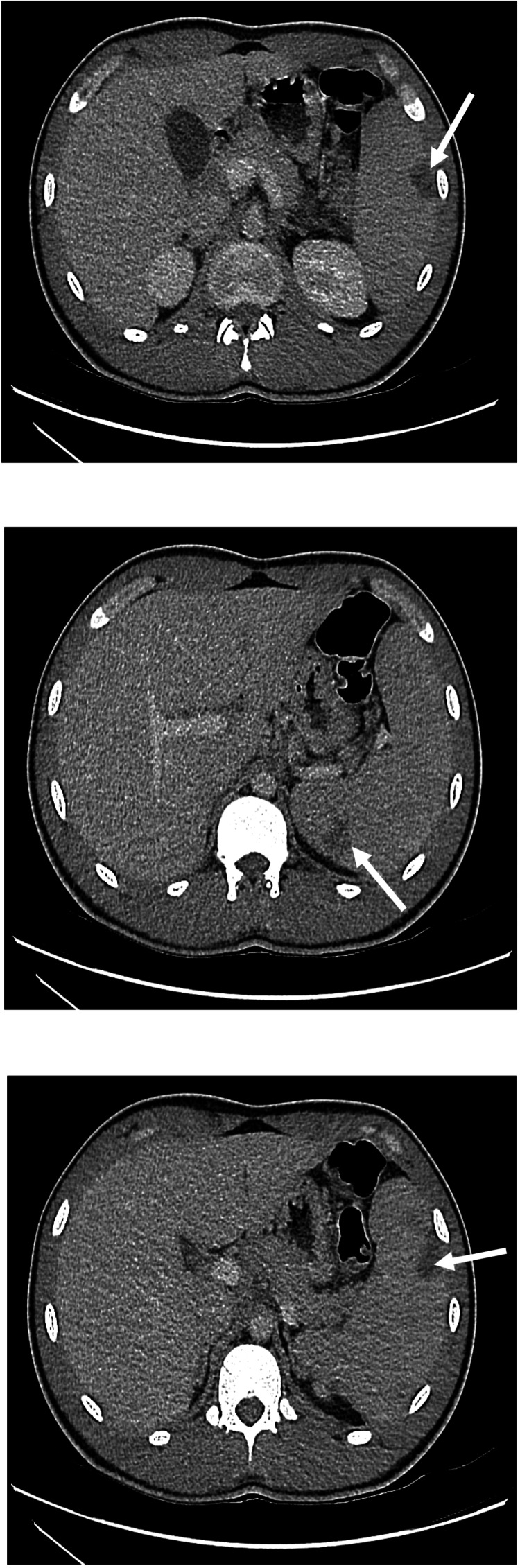
Cross-sectional imaging (axial view of computed tomography of the abdomen with intravenous contrast) demonstrating splenomegaly with hypoattenuated lesions indicative of splenic infarcts in the patient described (white arrows)

A computed tomography scan of the abdomen and pelvis with contrast revealed features of hepatosplenomegaly (the spleen measured 14 cm in craniocaudal length), with multiple wedge-shaped, low-density areas in the spleen most likely due to splenic infarcts (Figure 1) and a trace of fluid in the pelvis, but no evidence of colitis. Ultrasonography of the abdomen with Doppler confirmed splenic infarcts and splenomegaly at 16 cm in craniocaudal measurement.

He was admitted, and his azathioprine was suspended. Intravenous fluids and piperacillin-tazobactam were commenced to cover for secondary bacterial infection. From the second day of admission to the sixth day, he had pyrexial episodes up to 39.2 ºC. Due to a gradually diminishing haemoglobin, with low transferrin saturation and low iron levels, he was commenced on oral iron replacement. His activated partial thromboplastin time remained raised (48.5 seconds at its maximum) with a low haptoglobin of <0.03 g/L. He had a lymphocytosis of 27.2 x109/L at its maximum and a monocytosis of 3.0 x109/L at its maximum. As his imaging was indicative of splenic infarcts but no thromboses, he was given prophylactic rather than therapeutic dose anticoagulation. He completed two weeks of aciclovir for his shingles due to his immunocompromised state.

A blood film was indicative of atypical lymphocytosis, but no underlying haematological disease. A diagnosis of EBV infectious mononucleosis was confirmed by positive viral capsid antigen IgM antibodies. His protein C level was reduced at 0.55 IU/ml (local region normal range 0.74-1.64 IU/ml) with a normal protein S of 0.84 IU/ml (local region normal range 0.74-1.20 IU/ml) and normal anti-thrombin III activity of 1.01 IU/ml (local region normal range 0.80-1.20 IU/ml). A direct antiglobulin test was positive due to C3d. Genotyping was positive for a heterozygous mutation of Factor V Leiden. Flow cytometry demonstrated expansion of CD8+ T lymphocytes and loss of CD7, supporting findings of EBV infection.

He had no clinical signs of infective endocarditis. His blood culture and urine culture were negative, and a transthoracic echocardiogram showed a trivial pericardial effusion and mild pulmonary regurgitation but was otherwise unremarkable, demonstrating no features of vegetation. Paracetamol and salicylate blood levels were undetectable. Cytomegalovirus polymerase chain reaction testing was negative. He was human immunodeficiency virus, Hepatitis A, Hepatitis B and Hepatitis C negative. His anti-nuclear antibody, anti-neutrophil cytoplasmic proteinase-3 antibody, anti-neutrophil cytoplasmic myeloperoxidase antibody, anti-cardiolipin IgG antibody, anti-cardiolipin IgM antibody, anti-beta-2-glycoprotein-1 IgG antibody, and anti-beta-2-glycoprotein-1 IgM antibody tests were negative. Toxoplasma gondii deoxyribonucleic acid levels were negative. Alpha-fetoprotein levels were negative.

His liver function tests and C-reactive protein level improved, and he was no longer pyrexial. He was discharged nine days after admission with a follow-up appointment in a week’s time to check his full blood count and liver function tests, and to conduct a further blood film. A repeat computed tomography scan of the abdomen was arranged to check for resolution of splenomegaly in eight weeks’ time. He was advised to avoid sharing secretions and to avoid boxing and football for at least six months due to the risk of splenic rupture. This was particularly challenging for the young man, who was a keen boxer who had fought in the boxing ring earlier and worked as a personal fitness trainer at a gym.

## Discussion

Infectious mononucleosis is usually a self-limiting illness, characterised by fever, pharyngitis and lymphadenopathy, which is managed with best supportive care. The diagnostic test with the greatest sensitivity for the commonest cause of infectious mononucleosis (Epstein-Barr virus) is viral capsid antigen IgM antibodies [1]. Despite splenomegaly being prevalent amongst patients with infectious mononucleosis, the presence of splenic infarcts is increasingly rare, with just 49 cases reported in the literature, which includes cases with concomitant predisposing haematological conditions [2]. Whether the incidence of splenic infarcts in infectious mononucleosis is expectedly low or whether it is artificially reduced by underdiagnosis remains equivocal [1].

Splenic infarcts following Epstein-Barr virus infection are usually hypothesised to occur due to ischaemia from rapid enlargement of the spleen or a shift towards a prothrombotic balance; however, their true pathophysiology remains indeterminate [2-4]. A transitory, induced reduction in the level of protein C has been implicated in the possible pathogenesis of splenic infarcts in infectious mononucleosis and was likely demonstrated in this case; however, whether this was a sufficiently significant factor has yet to be elucidated [1,4,5]. Conversely, splenic infarction has been evident in patients without hypercoagulability on a coagulation screen or co-morbidities [6]. Moreover, immunological mechanisms, including complement activation, are a further postulated aetiology [7].

Localising features that might prompt further investigation for possible splenic infarcts include tenderness or pain in the left upper quadrant abdominal region [8]. Contrast-enhanced computed tomography is the optimum imaging modality for identification of splenic infarcts; however, ultrasonography may also be used, particularly in children, to avoid radiation exposure, and less commonly, magnetic resonance imaging is also used [4,9,10]. Although a seemingly uncommon occurrence, when found, splenic infarcts should be closely monitored due to their risk of mortality from catastrophic exsanguination. Conservative management appears to be sufficient in the majority of cases of splenic infarcts associated with infectious mononucleosis [8,11,12]. Rarely, splenectomy may need to be performed in cases of haemodynamic instability or splenic rupture. No cases of mortality have been reported in the literature following splenic infarcts from Epstein-Barr virus, and splenic radiological findings and the associated prothrombotic states appear to regress following the infection [5]. Splenic infarcts from Epstein-Barr virus, whether considerably underdiagnosed or extraordinarily uncommon, warrant further investigation to clarify their pathogenesis. Importantly, counselling to avoid contact sports must be given to such patients to minimise the chance of splenic rupture [13].

## Conclusions

Splenic infarction is a rare but important complication of Epstein-Barr virus infectious mononucleosis. Clinicians should maintain vigilance when managing patients presenting with left upper quadrant pain in this cohort of patients, particularly in the context of splenomegaly. Early diagnosis allows appropriate monitoring and counselling to be implemented to prevent dangerous sequelae such as splenic rupture. Further research is required to elucidate the underlying pathophysiological mechanisms, including the role of transient hypercoagulability and splenic enlargement.
